# Clinic of the Chicago Dental Club

**Published:** 1887-06

**Authors:** Arthur B. Freeman

**Affiliations:** Secretary


### CLINIC OF THE CHICAGO DENTAL CLUB.

REPORTED FOR THE INDEPENDENT PRACTITIONER BY ARTHUR B. FREEMAN, M. D., D. D. S., SECRETARY.

At the March meeting of the club, it was voted that the afternoon of the day on which the regular monthly meeting is held (the fourth Monday of each month) be devoted to a clinic. In pursuance of this action the business committee arranged a clinic, which was held in the commodious office of Dr. Chas. Pruyn, No. 70 Dearborn Street, on Monday, April 25th, at 1 o'clock P. M.

Between forty and fifty members of the club and profession were in attendance. The following is a condensed report:

Dr. L. P. Haskell demonstrated the use of "cores" in securing dies of a difficult partial lower case, having a long, high ridge on the left side, with an undercut on both surfaces, so that it was wider at the crest than lower down. The method of making the core is simple, and in such cases of undercut it is indispensable in securing a perfect adaptation of the plate. Asbestos and plaster are mixed and run into the undercut on the plaster cast, trimmed, varnished, dried of moisture over the gas stove, and placed in position, when a mold in sand is secured, the cores readily dropping from the sand mold with the cast. They should now be replaced in the sand and the metal poured.

If the precaution of drying the core is not taken, the steam generated by the hot metal causes bubbling and imperfection of the die. The necessity of using asbestos in the cores, in preference to any other material, was demonstrated by the fact that the fiber had preserved it from breaking, the same cores having been twice used before in making a practical case, and were still in a condition for further use.

Dr. Eugene S. Talbot demonstrated, on plaster models, his method of reducing irregularities by the use of the piano-wire coil springs, and also his method of manufacturing them for individual cases, which was by placing an excavator in a vise, and closely bending the piano-wire about it several times.

Dr. J. H. Woolley exhibited his new root dryer, which is composed of a steel handle so adjusted to a copper cone or bulb, that the two act as a chuck to hold *in situ* a copper needle. The bulb is heated over a spirit lamp, and acts as a reservoir, conducting the heat through the needle into the root canal. He demonstrated the use of the instrument in the roots of an inferior first molar, and proved it to be a convenient and efficient acquisition to the operating table.

Dr. J. G. Reid, in the canals previously prepared by Dr. Woolley, demonstrated his method of root filling, which is that used by so many operators, the gutta-percha method,

Dr. E. R. Mullett, of Clinton, Iowa, exhibited his recently perfected electric mallet, and demonstrated its use by filling with gold a labial cavity in a lower incissor, which was partially under the margin of the gum.

Dr. A. E. Matteson filled with gold a large cavity in a superior molar, in which a fissure extended from the crown over the buccal surface toward the soft tissues.

His mallet was a unique instrument, consisting of a heavy leaden head, with a hole in which the index finger of the right hand fitted. By rapid action of the extensor and flexor muscles he caused the blows to fall upon a plugger held by the other fingers of the same hand, as effectively if not quite as rapidly as those of the electric mallet. The filling was quickly and beautifully done, and demonstrated that entire command of a few good instruments and methods is better than a constant vacillation among many.

Dr. J. Austin Dunn exhibited his new syringe for injecting remedies in alveolar abscesses, pyorrhœal pockets, root canals, etc. It consists of a rubber bulb, connected with a curved adjustable needle or point. It is a very light and convenient instrument, capable of delicate manipulation and at the same time equal in force to the hypodermic syringe.

Dr. McIntosh, of the McIntosh Galvanic and Faradic Battery Co., made an interesting and instructive exhibit of electrical instruments and microscopes suited to dental work.

An improved plunge battery, manufactured by his company after the designs of Dr. Geo. W. Whitefield, a member of the club, excited considerable interest. The elements are zinc and carbon, the latter stationary in the fluid, the former raised and lowered by the foot at will, giving perfect control of the current while leaving the hands free.

The McIntosh "Professional Microscope" was shown, with a device for illuminating the object by the electric light attached. The perfect definition secured appeared to surpass the best results obtainable from sunlight.

"The Doctor's Throat and Mouth Mirror," lighted by a small incandescent lamp, was pronounced exceedingly convenient. As an aid in examinations it will become almost indispensable. A new instrument that excited special interest, is designed for drying nerve cavities in the teeth preparatory to filling, and was also suggested by Dr. Whitefield. It is constructed on the principle of the galvano-cautery electrode, and is heated by the electric current. The heat is under perfect control, the instrument being introduced cold and heated *in situ* to any degree wanted, instantly. Cutting off the current allows it to cool almost as rapidly as it is heated.

Dr. McIntosh has recently perfected a new bracket, suggested by Dr. Whitefield, having a revolving arbor connected by a belt with his new electric motor, both of which were shown to the dentists present. The arbor extends from the wall about three feet, and has attached to the end a flexible arm and hand-piece, any of the arms or hand-pieces used on dental engines being readily adjustable.

Worthy of notice is the new Universal Dental Matrix, the invention of Dr. Jno. H. Reed, of Lancaster, Wis., which was exhibited. This matrix has at least one redeeming feature which most do not possess, namely, its trifling cost. The advantages claimed are perfect adaptability, simplicity, durability and economy.

In addition to the foregoing, slides showing the invagination of the epithelium in the early embryonic development of the teeth were shown through the two fine microscopes on exhibition. The Electric and the Shaw Dental Engines were also exhibited.

A dozen patients were present for examination and consultation, with cleft palates, irregularities of the teeth, and other deformities of a nature requiring great judgment to correct or replace with artificial substitutes.

At six o'clock those in attendance adjourned to the Tremont House, where they dined together, after which the club held its regular monthly meeting, and after the transaction of its business the members discussed and criticised the implements exhibited and the operations performed.

After the discussion Dr. McIntosh briefly addressed the club on Electricity, and before closing his remarks invited the members to meet in special session, at some future date, at the exhibit rooms of his company, when he said he would be glad to show their more delicate electrical apparatus and give them a more detailed talk on the subject, as well as to show them some curious experiments.

When the club adjourned at ten o'clock the general verdict was that a very profitable afternoon had been spent together, and an enthusiasm prevailed which we hope to see continued.

